# Beneficial Effects of *Eruca sativa* Defatted Seed Meal on Visceral Pain and Intestinal Damage Resulting from Colitis in Rats

**DOI:** 10.3390/foods11040580

**Published:** 2022-02-17

**Authors:** Elena Lucarini, Laura Micheli, Eleonora Pagnotta, Roberto Matteo, Carmen Parisio, Alessandra Toti, Valentina Ferrara, Clara Ciampi, Alma Martelli, Lara Testai, Vincenzo Calderone, Michele Savino, Mario Russo, Nicola Pecchioni, Carla Ghelardini, Lorenzo Di Cesare Mannelli

**Affiliations:** 1 Department of Neuroscience, Psychology, Drug Research and Child Health (NEUROFARBA), Pharmacology and Toxicology Section, University of Florence, 50139 Florence, Italy; elena.lucarini@unifi.it (E.L.); laura.micheli@unifi.it (L.M.); carmen.parisio@unifi.it (C.P.); alessandra.toti@unifi.it (A.T.); valentina.ferrara@unifi.it (V.F.); clara.ciampi@stud.unifi.it (C.C.); carla.ghelardini@unifi.it (C.G.); 2 CREA-Council for Agricultural Research and Economics, Research Centre for Cereal and Industrial Crops, 40128 Bologna, Italy; eleonora.pagnotta@crea.gov.it (E.P.); roberto.matteo@crea.gov.it (R.M.); 3 Department of Pharmacy, University of Pisa, 56126 Pisa, Italy; martelli@farm.unipi.it (A.M.); lara.testai@farm.unipi.it (L.T.); vincenzo.calderone@unipi.it (V.C.); 4 Interdepartmental Research Centre Nutraceuticals and Food for Health (NUTRAFOOD), University of Pisa, 56126 Pisa, Italy; 5 Interdepartmental Research Centre of Ageing Biology and Pathology, University of Pisa, 56126 Pisa, Italy; 6 CREA-Council for Agricultural Research and Economics, Research Centre for Cereal and Industrial Crops, 71122 Foggia, Italy; michele.savino@crea.gov.it (M.S.); mario.russo@crea.gov.it (M.R.); nicola.pecchioni@crea.gov.it (N.P.)

**Keywords:** inflammatory bowel diseases, *Brassicaceae*, glucosinolates, H_2_S, Kv7 potassium channel, mast cell, enteric nervous system

## Abstract

Most therapies used in patients affected by inflammatory bowel diseases are ineffective in preventing the development of chronic visceral hypersensitivity, mainly due to inflammation-induced enteric neuroplasticity. Glucosinolates, secondary metabolites mainly of *Brassicaceae* with anti-inflammatory and neuroprotective properties, are effective in treating both neuropathic and arthritis pain through H_2_S release and Kv7 potassium channel activation. The aim of this work was to investigate the protective and anti-hyperalgesic efficacy of a defatted seed meal from *Eruca sativa* Mill. (Brassicaceae), rich in glucosinolates, in a rat model of colitis induced by 2,4-dinitrobenzene sulfonic acid (DNBS). The mechanisms of action were also investigated. Visceral pain was assessed by measuring the abdominal response to colorectal distension. Fifteen days after colitis induction, the acute administration of *E. sativa* defatted seed meal (0.1–1 g kg^−^^1^ p.o.) dose-dependently relieved pain. This effect was hampered by co-administering an H_2_S scavenger or a selective Kv7 blocker. Administering *E. sativa* (1 g kg^−1^) for 14 days, starting after DNBS injection, contributed to counteracting visceral pain persistence in the post-inflammatory phase of colitis by promoting colon healing from the damage and reducing enteric gliosis. *E. sativa* defatted seed meal might be employed as a nutraceutical tool for supporting abdominal pain relief in patients.

## 1. Introduction

The management of patients with inflammatory bowel diseases (IBDs) still represents a therapeutic problem. In most cases, the chronic inflammatory processes lead to the establishment of a functional gastrointestinal disease (characterized by pain, bloating, and dysmotility), which persists after the remission of colitis, making it difficult to treat [1,2]. The complex nature of this type of disease, both inflammatory and neuropathic, makes patients partially responsive to anti-inflammatory drugs, spasmolytics, anticonvulsants, or antidepressants, which are classically used in the management of other pain conditions [1,3].

In the search for novel therapies, increasing attention is paid to compounds, either natural or synthetic, able to exert combined anti-inflammatory and neuroprotective effects in vivo. Glucosinolates (GSLs), phytochemicals peculiar to *Brassicaceae*, and their hydrolysis-derivatives, isothiocyanates (ITCs), are among the most promising naturally occurring molecules in the treatment of inflammatory and neurological disorders, such as pain [4,5,6,7]. The biological effect of GSLs and their by-products is the result of a series of molecular mechanisms acting simultaneously, such as the modulation of xenobiotic metabolism and the regulation of inflammatory response, apoptosis, and oxidative stress. Moreover, recent evidence has attested to the ability of GSLs and ITCs to modulate the activity of channels, namely, Kv7 potassium channels, and receptors involved in pain transmission [4,6].

The pathways modulated by GSLs and ITCs are also relevant in the pathophysiology of visceral hypersensitivity. Broccoli sprouts and their characteristic ITC sulforaphane displayed significant spasmolytic and antinociceptive activities in a mice experimental model of abdominal pain involving the μ opioid receptors [8]. It is also acknowledged that most GSLs can behave as H_2_S donors in vivo [4,6]. Interestingly, treating rats with NaHS or H_2_S releasers resulted in a dose-dependent attenuation of pain induced by colorectal distension [9,10,11]. Despite the wide range of beneficial effects endowed by GSLs on painful diseases and the fact that most of the *Brassicaceae* are edible plants, the therapeutic potential of *Brassicaceae*-based foods in the management of chronic pain originating from the gut, as in the case of colitis, has not been explored yet.

Franco et al. developed a food-safe organic material to employ in the production of functional foods, starting from *Eruca sativa* (Eruca sativa Mill. Sel. NEMAT), a plant belonging to the *Brassicaceae* with a high content of GSLs in seeds, which was selected as an industrial oleaginous crop ready to be cultivated by conventional agricultural mechanization [12]. Notably, the pressure defatted oilseed meal (DSM) developed by Franco et al. resulted in being enriched with GSLs [13]. Once administered in a preclinical model of diabetic neuropathy, *E. sativa DSM* evoked a dose-dependent relief of thermal allodynia and mechanical hyperalgesia, through the release of H_2_S and the opening of Kv7 potassium channels, in a completely analogous way to the isolated GSL, glucoerucin [4].

The present work is aimed at evaluating the therapeutic potential of *E. sativa* DSM in the management of the persistent visceral pain resulting from inflammatory damage to the colon. Colitis induced by 2,4-dinitrobenzenesulfonic acid (DNBS) injection in rats is indeed associated with the development of a visceral hypersensitivity that persists after colitis remission because of the establishment of concomitant alterations in the immune response, microbiota metabolism, nerve signaling, and glia function both in animals and humans [14,15,16,17].

## 2. Materials and Methods

### 2.1. Animals

The experiments were conducted on three-month-old male Sprague-Dawley rats (Envigo, Varese, Italy) approximately weighing 220–250 g. The animals were housed in cages 26 × 41 cm (4 rats per cage) at CeSAL (Centro Stabulazione Animali da Laboratorio, University of Florence) and were maintained on a 12-h light/dark cycle with controlled environmental temperature (23 ± 1 °C) and free access to food and water. Animal manipulations were carried out according to the Directive 2010/63/EU of the European Parliament and of the European Union Council (22 September 2010) on the protection of animals used for scientific purposes. The ethical policy of the University of Florence complies with the Guide for the Care and Use of Laboratory Animals of the US National Institutes of Health (NIH Publication No. 85-23, revised 1996; University of Florence assurance number: A5278-01). Formal approval to conduct the described experiments was obtained from the Animal Subjects Review Board of the University of Florence and by the Italian Ministry of Health (543/2017-PR). Experiments involving animals have been reported according to ARRIVE guidelines [18]. All efforts were made to minimize animal suffering and to reduce the number of animals used.

### 2.2. Induction of Colitis

Colitis was induced as previously described [14]. During a brief anesthesia with isoflurane (2%), animals were intrarectally injected with 2,4-dinitrobenzenesulfonic acid (DNBS; Sigma-Aldrich, Italy; 20 mg in 0.25 mL of 50% ethanol) 8 cm proximal to the anus by a polyethylene PE-60 catheter. Control rats received 0.25 mL of saline solution.

### 2.3. Eruca Sativa Characterization

*Eruca sativa* DSM was produced according to the method described [4], while myrosinase (β-thioglucoside glucohydrolase, EC 3.2.1.147) was obtained from *Sinapis alba* seeds as previously reported [19] and stored (38 U/mL stock solution) at 4 °C. MYR activity, expressed in unit (U), was defined as the amount of enzyme able to hydrolyze 1 μmol of sinigrin/min at pH 6.5 and 37 °C. Glucosinolates content in *E. sativa* DSM was determined as previously described [4], while total polyphenols (TPC) and total flavonoids (TFC) were measured by spectrophotometric methods using a double-beam high-performance UV/VIS PC spectrophotometer (Lambda 25; PerkinElmer SpA; Waltham, MA, USA). Extraction of soluble conjugated compounds was performed as follows: 500 mg of whole *E. sativa* DSM were extracted twice with 5 mL methanol/1N HCl (85:15) solution by 20 min sonication, with nitrogen gas introduced into the tubes, and centrifugation (4500 rpm, 10 min, 4 °C). Supernatants were recovered and stored at −20 °C to precipitate the large molecules. 5 mL of the methanolic extracts were digested with 6 mL 4N NaOH in sonication bath for 10 min. The solutions were then brought to pH 2 with 6 M HCl and extracted twice with 10 mL of diethyl ether/ethyl acetate (1:1 *v*/*v*). The extracts were clarified for centrifugation, evaporated, resuspended in 2 mL methanol/H_2_O (80/20 *v*/*v*), filtered with a 0.22 µm PTFE filter and then analyzed. The insoluble bound phenolics were obtained from the solid residue of methanolic extracts and were further digested in 12 mL 4N NaOH in sonication bath for 30 min, vortexing every 5 min. After centrifugation, supernatant was brought to pH 2 with 6 N HCl and extracted twice with 10 mL of diethyl ether/ethyl acetate (1:1 *v*/*v*). After separation, the organic phase was clarified for centrifugation, evaporated, resuspended in 2 mL methanol/H_2_O (80/20 *v*/*v*), filtered with a 0.22 µm polytetrafluoroethylene (PTFE) and analyzed by spectrophotometer. TPC was analyzed following Gao et al. [20] using the Folin–Ciocalteu method. In brief, 200 µL of methanolic extracts were diluted with 2.6 mL water, then 200 µL of 0.2 M Folin–Ciocalteu reagent were added, and the mixture was allowed to stand for 3 min at room temperature. Then 900 µL of 1 M Na2CO3 were added, and after 90 min of reaction at room temperature, the absorbance was determined at 765 nm. The absorbance values were compared to the standard curve of gallic acid (10–100 µg/mL) and TPC was expressed as gallic acid equivalents (GAE; µg g^−1^).

TFC was determined according to Zhishen et al. [21], 1 mL of extract was diluted in 4 mL of distilled water and mixed with 0.3 mL of 5% (*w*/*v*). After five minutes, 0.3 mL of 10% (*w*/*v*) AlCl3 was added. After 6 min, 2 mL of 1 N NaOH were added, and the volume was made up to 10 mL with distilled water. The solution was mixed well and allowed to stand for 15 min before reading the absorbance at 510 nm. The TFC was calculated from a calibration curve (10–150 µg mL^−1^), and the results were expressed as mg of catechin equivalent per g of *E. sativa* DSM.

### 2.4. Experimental Design and Treatments

In the first experimental set the acute effect of *E. sativa* DSM on visceral hypersensitivity induced by colitis was investigated. Fourteen days after DNBS injection, the animals were orally administered with *E. sativa* DSM or the vehicle. For the administration, *E. sativa* DSM was suspended in phosphate-buffered saline (PBS) and orally administered to the animals by *gavage*. The doses of *E. sativa* DSM (0.1–1 g kg^−1^) were chosen based on previous evidence [4]. In a group of animals, *E. sativa* DSM was bioactivated by adding, 15 min before the administration, 30 μL mL^−1^ of myrosinase (38 U mL^−1^) to its PBS suspension, which resulted in an 85% glucoerucin-erucin conversion rate, as evaluated in gas chromatography by using benzyl isothiocyanate as internal standard. In a group of animals, the most effective dose of *E. sativa* DSM (1g kg^−1^) was administered in mixture with glutathione (GSSG) 65 μmol kg^−1^ (20 mg kg^−1^; GSSG; Sigma-Aldrich, Milan, Italy). In another group, the Kv7 potassium channel blocker XE991 (Tocris Bioscience, Italy; 2.66 μmol kg^−1^; 1 mg kg^−1^; [22]), dissolved in saline solution, was intraperitoneally administered concurrently with *E. sativa* DSM oral administration. In each experimental subset, a group of controls (reported as “vehicle”) and a group of DNBS animals (reported as “DNBS + vehicle”) received an oral gavage of the vehicle used for the preparation of *E. sativa* DSM. All behavioral tests were carried out 30 min after the administration of *E. sativa* DSM.

In the second experimental set, the effect of the repeated administration of *E. sativa* DSM in DNBS-treated animals was investigated. Repeated oral administrations of *E. sativa* DSM (1 g kg^−1^) were carried out for 14 days, from day 0 to day 13 after damage induction. The group of controls (reported as “vehicle”) and a group of DNBS animals (reported as “DNBS + vehicle”) received an oral gavage of the vehicle used for the preparation of *E. sativa* DSM. Behavioral tests were performed on days 7 and 14, 24h after the last treatment. Once the tests were completed, the animals were sacrificed, and colon tissue was collected for histological analysis.

### 2.5. Assessment of Visceral Sensitivity by Abdominal Withdrawal Reflex to Colorectal Distension

To perform colorectal distension, a balloon (length: 4.5 cm) tightened to an embolectomy catheter and connected with a syringe was inserted into the colon of animals undergoing light anaesthesia (2% isoflurane). The external part of the catheter was then fixed to the tail. During the measure, the balloon was filled with increasing volumes of water (0.5, 1, 2, and 3 mL; 5 min was the time elapsed between two consecutive distensions). A semi-quantitative score (0 to 4) was assigned to animal’s abdominal withdrawal response (AWR) as described previously [14,23].

### 2.6. Assessment of Colon Damage

Colon macroscopic and microscopic damage were assessed in accordance with the criteria reported previously [24]. Colon segments were fixed in 4% paraformaldehyde for 24 h, dehydrated in alcohol, embedded in paraffin, and finally cut into 5 μm sections. Microscopic evaluations were carried out on haematoxylin-eosin stained sections of full-thickness samples obtained from the distal colon. GIEMSA staining (Sigma-Aldrich, Milan, Italy) was used to analyse mast cells (MCs) density (cell number/respective arbitrary field) in the submucosal layer of colon [25]. Digitalized images were collected at 40× magnification by a Leica DMRB light microscope, equipped with a DFC480 digital camera (Leica Microsystems, Milan, Italy), and analysed quantitatively using the ImageJ software. At least five independent arbitrary optical fields (0.1 mm^2^) were analysed for each animal.

### 2.7. Immunofluorescence Analysis

For immunoreactions, tissue was cut into 5 µm slices and dried on glass slides prior to deparaffinization with xylol and rehydration in a descending alcohol series (100, 95, 75, and 50%). Tissues were rinsed in PBS containing 0.1% Triton X-100 (T-PBS), followed by a 1 h incubation in blocking solution (containing 0.1% Triton X-100 and 5% bovine serum albumin in 1X PBS) at room temperature. The slices were immunolabeled with a mouse anti-UCH-L1/PGP9.5 (Novus Biologicals-31A3, Bio-Techne Ltd., Abingdon, UK), diluted 1:500 in T-PBS/5% BSA (Sigma-Aldrich, Milan, Italy) and a rabbit anti-glial fibrillary acidic protein (GFAP, DAKO-Z0334, Agilent Technologies Italia, Milan, Italy), diluted 1:500 in PBS/5% BSA (Sigma-Aldrich, Milan, Italy). To stain nuclei, sections were incubated with DAPI Fluoromount-G™ Mounting Medium (Thermo Fisher Scientific, Milan, Italy) was used as mounting medium. Digitalised images were collected at 400× total magnification by a motorized Leica microscope DM6 B equipped with DFC9000 GT camera, supported by a THUNDER Workstation 3D DCV and by the software LAS X (Leica Biosystems, Milan, Italy). Quantitative analysis of PGP9.5- and GFAP-related immunofluorescence intensity (arbitrary unit) was performed by collecting independent fields (4–6 for each animal) from the myenteric plexi and analysing them by ImageJ (NIH, Bethesda, MD, USA). Results were expressed as a percentage of the control group (vehicle-treated animals).

### 2.8. Statistics

All the experimental procedures were performed by a researcher blind to the treatment. Results were expressed as mean ± SEM of 6 animals *per* group. The analysis of variance (ANOVA) was performed by one-way ANOVA with Bonferroni’s significant difference procedure used for post-hoc comparisons. *p* values of less than 0.05 were considered significant. Data were analyzed using the “Origin 9” software (OriginLab, Northampton, MA, USA).

## 3. Results

### 3.1. Eruca Sativa Defatted Seed Meal Characterization

Oil seed meal extraction was carried out by a mechanical, food-grade process, avoiding high temperatures and the use of solvents to preserve DSM quality. The chemical profile of *E. Sativa* DSM is reported in Table 1.

The main GSL in *E. sativa* DSM, glucoerucin (GER), accounts for 94.5% of total GSLs (128 μmol g^−1^). TPC and TFC content, both in their soluble and insoluble forms, account for 12.3 mg of GAE g^−1^ and 4.7 mg of CE g^−1^, respectively, with unbound molecules accounting for 89% of total compounds.

### 3.2. The Acute Administration of Eruca Sativa DSM Relieved Visceral Pain Induced by DNBS in Rats by the Release of H_2_S and the Activation of Kv7 Potassium Channels

**Figure 1:**
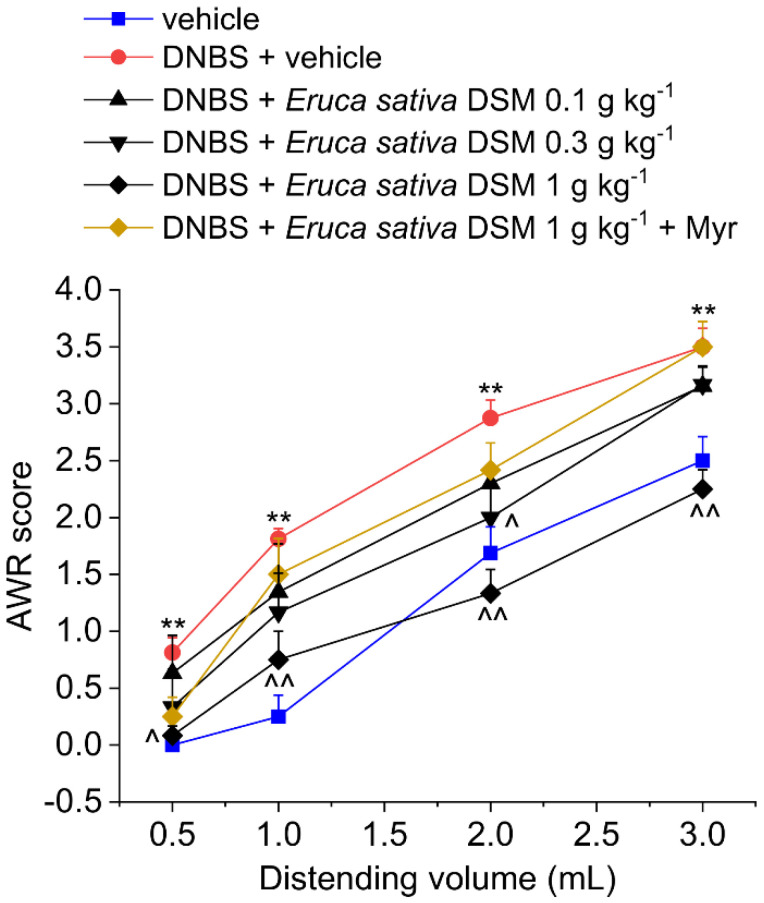
The effect of *Eruca sativa* DSM acute administration on post-inflammatory visceral pain caused by colitis in rats before and after bio-activation with myrosinase enzyme (Myr). Visceral sensitivity was assessed in animals by measuring the extent of the abdominal withdrawal response (AWR) to colorectal distension, carried out by applying an increasing distending stimulus on the colon walls (0.5–3 mL). The test was performed 30 min after the *E. sativa* DSM oral administration. Each value represents the mean ± SEM of six animals per group. ** *p* < 0.01 vs. vehicle. ^ *p* < 0.05 and ^^ *p* < 0.01 vs. DNBS + vehicle.

The acute effect of *E. sativa* DSM on post-inflammatory visceral pain was evaluated 14 days after DNBS injection (Figure 1) when visceral pain persists despite tissue healing [14]. Visceral pain was monitored in the animals by assigning a score to their abdominal withdrawal response (AWR) to colorectal distension (CRD). Fourteen days after colitis induction, the AWR’s 1–3 mL distending volume was significantly greater in DNBS-treated animals compared to the vehicle-treated group (Figure 1; *p* < 0.01 for each distending volume tested). The acute administration of *E. sativa* DSM (0.1–1 g kg^−1^ p.o.) dose-dependently relieved the visceral hypersensitivity induced by DNBS in rats. In particular, the dose of 1 g kg^−1^ significantly reduced animals’ AWR response to CRD back to the value of controls (vehicle treated group; Figure 1; *p* < 0.05 for 0.5 mL and *p* < 0.01 for 1–3 mL). *E. sativa* DSM 0.3 g kg^−1^ was partially effective, significantly reducing the AWR only at the stimulus of 2 mL (*p* < 0.05), while the effect elicited by the lower dose (0.1 g kg^−1^) did not reach statistical significance (Figure 1). *E. sativa* DSM anti-hyperalgesic efficacy was strongly reduced when pre-incubated with the myrosinase (thioglucosidase, EC 3.2.1.147; Myr), the enzyme responsible for the hydrolysis of GSLs (Figure 1).

**Figure 2:**
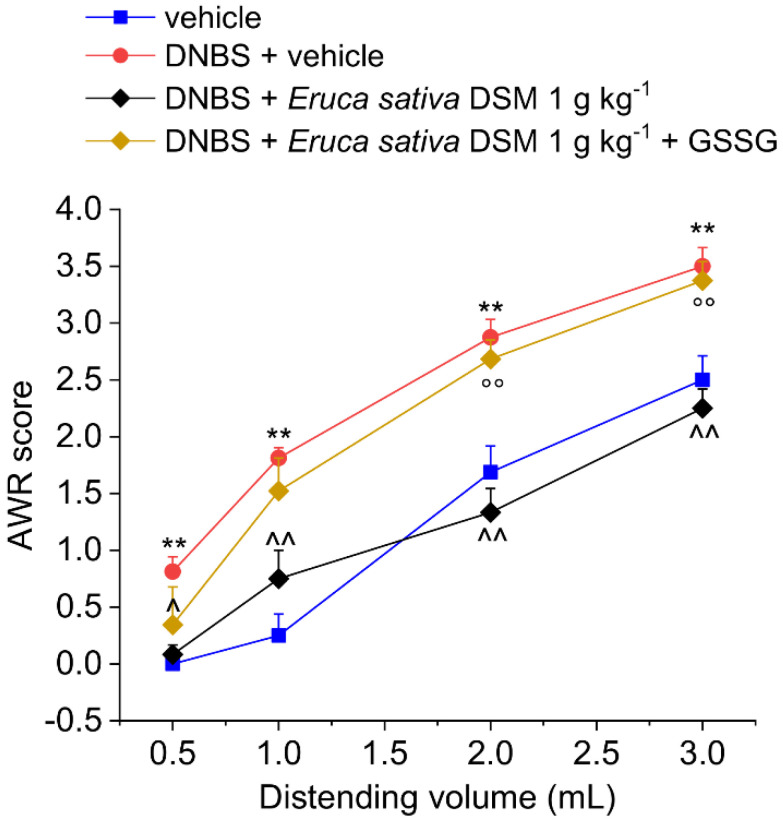
Role of H_2_S in the acute pain-relieving effect of *Eruca sativa* DSM. Visceral sensitivity was assessed in animals by measuring the extent of the abdominal withdrawal response (AWR) to colorectal distension, carried out by applying an increasing distending stimulus on the colon walls (0.5–3 mL). Oxidized glutathione (GSSG) (20 mg kg^−1^) was orally administered in concomitance with *Eruca sativa* DSM (1 g kg^−1^), and the test was performed after 30 min. Each value represents the mean ± SEM of six animals per group. ** *p* < 0.01 vs. vehicle. ^ *p* < 0.05 and ^^ *p* < 0.01 vs. DNBS + vehicle. °° *p* < 0.01 vs. DNBS + *Eruca sativa* DSM.

**Figure 3:**
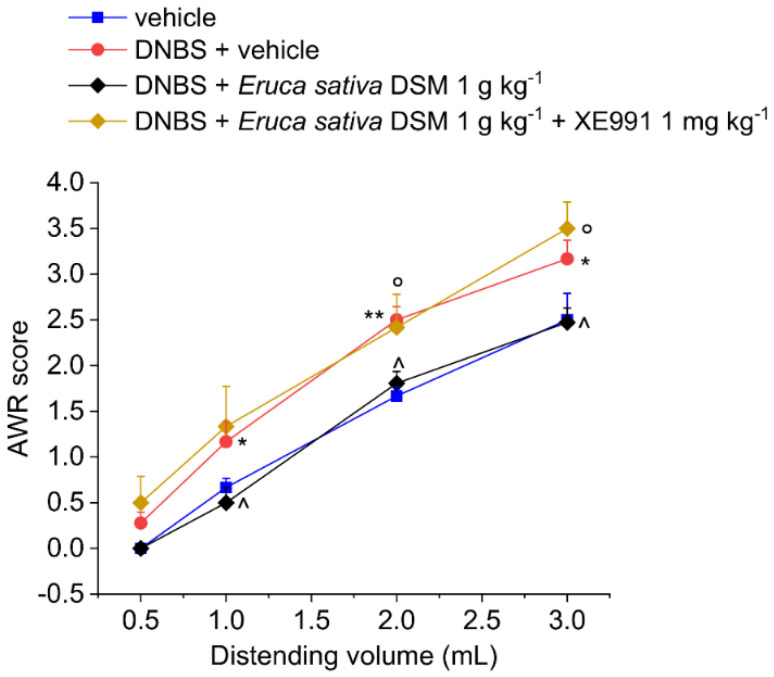
Involvement of the Kv7 potassium channels in the acute pain-relieving effect of *Eruca sativa* DSM. Visceral sensitivity was assessed in animals by measuring the extent of the abdominal withdrawal response (AWR) to colorectal distension, carried out by applying an increasing distending stimulus on the colon walls (0.5–3 mL). The Kv7 potassium channel blocker, XE991 (1 mg kg^−1^) was intraperitoneally administered in concomitance with *Eruca sativa* DSM (1 g kg^−1^), and the test was performed after 30 min. Each value represents the mean ± SEM of six animals per group. * *p* < 0.05 and ** *p* < 0.01 vs. vehicle. ^ *p* < 0.05 vs. DNBS + vehicle. ° *p* < 0.05 vs. DNBS + *Eruca sativa* DSM.

The acute pain relief mediated by *E. sativa* DSM in DNBS-treated animals was prevented by administering the H_2_S scavenger glutathione disulfide (GSSG) in a mixture with the meal (Figure 2; *p* < 0.01 with 2–3 mL) or by blocking the activation of Kv7 potassium channels with XE991 (1 mg kg^−1^ i.p.) (Figure 3; *p* < 0.05 with 2–3 mL).

### 3.3. The Repeated Treatment with Eruca Sativa DSM Counteracted Visceral Pain Persistence Induced by DNBS Colitis in Rats

**Figure 4:**
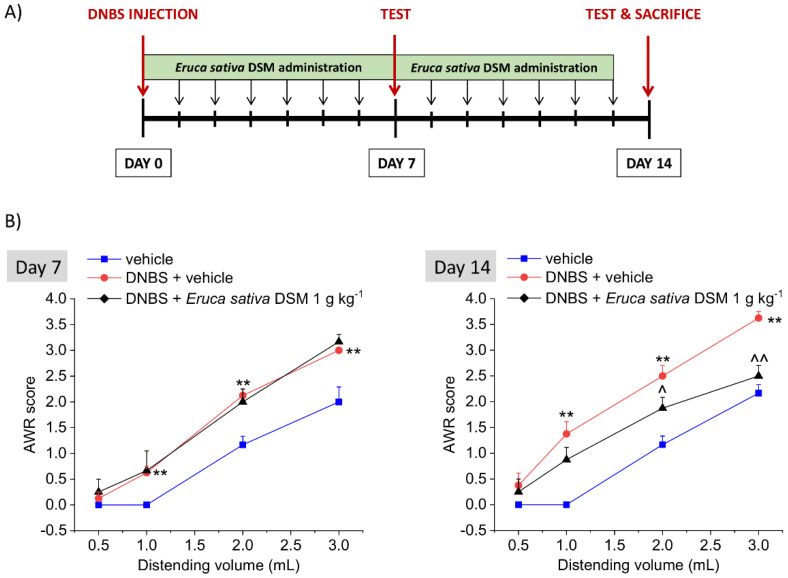
The effect of the repeated treatment with *Eruca sativa* DSM on visceral pain induced by colitis in rats. Experimental scheme (**A**); Visceral sensitivity was assessed in animals by measuring the extent of the abdominal withdrawal response (AWR) to colorectal distension, carried out by applying an increasing distending stimulus on the colon walls (0.5–3 mL). *Eruca sativa* DSM (1 g kg^−1^) was administered once daily in the DNBS-treated animals, starting from the day of DNBS injection for 14 consecutive days and pain threshold was assessed on day 7 (acute inflammatory phase) and 14 (post-inflammatory phase) (**B**). Each value represents the mean ± SEM of *6* animals per group. ** *p* < 0.01 vs. vehicle. ^ *p* < 0.05 and ^^ *p* < 0.01 vs. DNBS + vehicle.

*Eruca sativa* DSM (1 g kg^−1^ p.o.) was administered for 14 days, starting from the day of the DNBS injection. Visceral pain was assessed in the acute inflammatory phase (day 7) and in the post-inflammatory phase (day 14) of colitis, 24 h after the last administration of the compounds, by measuring the abdominal withdrawal response (AWR) to colorectal distension (CRD) (experimental scheme; Figure 4A). As a result of the colitis induced by DNBS, the animals developed a visceral hypersensitivity, as attested by the increased AWR response to CRD with respect to controls (vehicle group; Figure 4B, day 7; *p* < 0.01 with 1–3 mL), also in the remission phase of colitis (Figure 4B, day 14; *p* < 0.01 with 1–3 mL).

The treatment with *E. sativa* DSM (1 g kg^−1^) significantly counteracted the consolidation of visceral hyperalgesia in the post-inflammatory phase of colitis induced by DNBS (Figure 4B, day 14), though it was ineffective in preventing the establishment of pain caused by the acute inflammatory damage in the same animals (Figure 4B, day 7). Indeed, on day 14, animals receiving DNBS + *E. sativa* DSM showed a significantly lesser AWR than those treated with DNBS + vehicle when 2- and 3-mL stimuli were applied (Figure 4B, day 14; *p* < 0.05 with 2 mL and *p* < 0.01 with 3 mL), while no difference were observed between DNBS animals receiving the meal or the vehicle on day 7 (Figure 4B).

### 3.4. The Repeated Treatment with Eruca Sativa DSM Promotes Tissue Healing after Colitis Induced by DNBS

**Figure 5:**
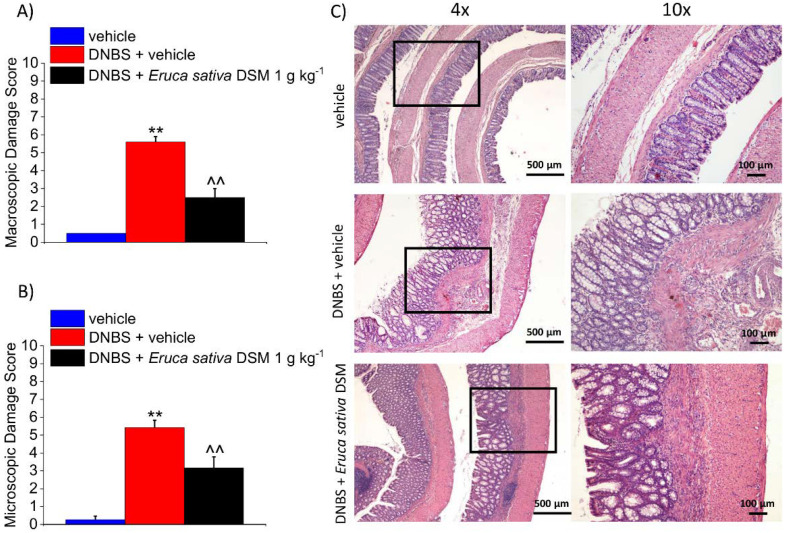
The effect of the repeated treatment with Eruca sativa DSM on colon damage induced by DNBS in rats. Eruca sativa DSM (1 g kg^−1^) was administered once daily in the DNBS-treated animals, starting from the day of DNBS injection for 14 consecutive days, then tissues were collected. Colon macroscopic (**A**) and microscopic (**B**) damage score; Representative pictures of haematoxylin-eosin stained sections of full-thickness colon (**C**). Original magnification: 4× and 10×. Each value represents the mean ± SEM of 6 animals per group. ** *p* < 0.01 vs. vehicle. ^^ *p* < 0.01 vs. DNBS + vehicle.

The animals were sacrificed 14 days after the DNBS injection. The colon was analyzed both macroscopically (Figure 5A) and microscopically (Figure 5B) to assess the damage. In the post-inflammatory phase of colitis, significant alterations in the colon histology of DNBS-treated animals are still present (*p* < 0.01 with respect to the vehicle group). Despite the restoration of the tunica mucosa, the colon of DNBS-treated animals appeared significantly thickened with residual inflammatory infiltrate, irregular shaped crypts, and goblet cell hyperplasia responsible for mucus hypersecretion (Figure 5C). The repeated treatment with *E. sativa* DSM was able to significantly reduce the damage induced by DNBS, as attested by significantly lower macroscopic and microscopic damage scores with respect to the DNBS + vehicle group (Figure 5A, B, respectively; *p* < 0.01). As a result of *E. sativa* DSM treatment, the tunica mucosa of DNBS animals was mostly restored and their crypts showed a structure and shape similar to those of the controls, though an inflammatory infiltrate was still present in the submucosa (Figure 5C).

### 3.5. The Repeated Treatment with Eruca Sativa DSM Reduced Mast Cell Infiltration and Enteric Glia Activation Resulting from Colitis in Rats

**Figure 6:**
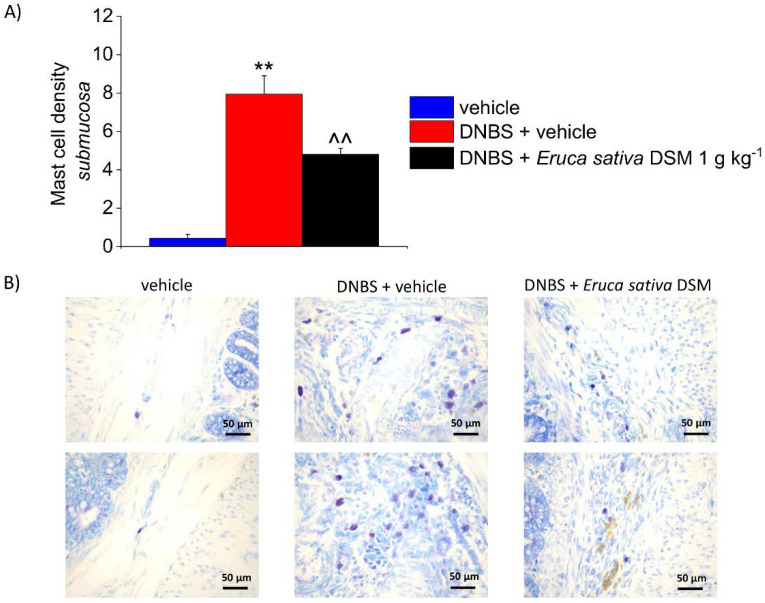
The effect of the repeated treatment with *Eruca sativa* DSM on submucosal MCs increase, induced by DNBS. *Eruca sativa* DSM (1 g kg^−1^) was administered once daily in the DNBS-treated animals, starting from the day of DNBS injection for 14 consecutive days, then tissues were collected. Column graphs display the mean values of MCs density per area of colonic wall (cells/field) (**A**). The panel shows pictures captured from submucosa of MCs stained with GIEMSA (**B**). Each value represents the mean ± SEM of *six* animals per group. ** *p* < 0.01 vs. vehicle. ^^ *p* < 0.01 vs. DNBS. Original magnification: 40×.

According to previous evidence collected in both human and animals, visceral pain is correlated with an augmented activity of MCs through the intestine [14,26]. In the post-inflammatory phase of colitis induced by DNBS, an increased density of MCs has been detected in the submucosa with respect of controls (*p* < 0.01), accompanying pain persistence. As a result of *E. sativa* DSM repeated administration, the number of MCs detected in the colon mucosa of DNBS-treated animals was significantly lowered (Figure 6A; *p* < 0.01). The reduced mast cell density clearly emerges from the illustrative images in Figure 6B.

In the gut, another important actor involved in the maintenance of local homeostasis is the enteric glia, which continuously controls what is happening in the surrounding environment and responds accordingly [27,28,29,30].

**Figure 7:**
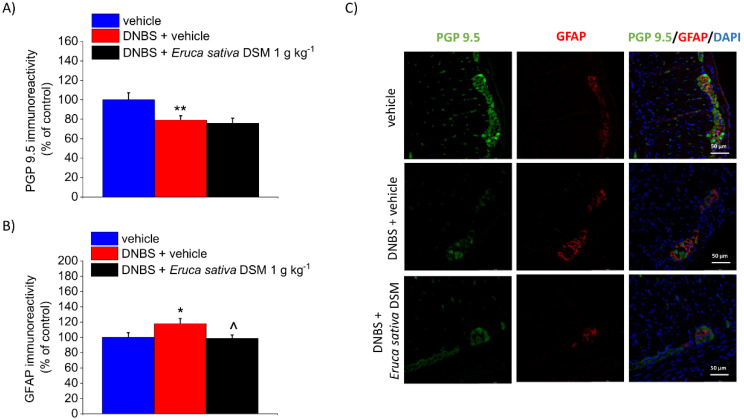
The effect of the repeated treatment with *Eruca sativa* DSM on neuronal damage and glia activation within the colonic myenteric plexus of DNBS rats. *Eruca sativa* DSM (1 g kg^−1^) was administered once daily in the DNBS-treated animals, starting from the day of DNBS injection for 14 consecutive days, then tissues were collected. Immunolabeling quantification of PGP 9.5 (**A**) and GFAP (**B**) with relative immunofluorescence images showing the expression of PGP 9.5 (green), GFAP (red), and DAPI (blue) in the myenteric plexus of the colon (**C**). Quantitative analysis of PGP9.5- and GFAP-related immunofluorescence intensity (arbitrary unit) was performed by collecting independent fields (4–6 for each animal) from the myenteric plexi. Results were expressed as a percentage of the control group (vehicle-treated animals). Each value represents the mean ± SEM of *six* animals per group. * *p* < 0.05 vs. vehicle. ** *p* < 0.01 vs. vehicle. ^ *p* < 0.05 vs. DNBS. Original magnification: 40×.

Enteric glia activation can be detected by changes in the marker’s expression, such as GFAP, reflecting altered physiological functions and potential deleterious effects on neuronal cells [31,32,33]. DNBS has been reported to trigger an enteric glia-mediated inflammatory response in the colon [34], so we investigated whether *E. sativa* DSM might antagonize these changes. In the post-inflammatory phase of colitis (Day 14), immunofluorescence analysis performed on the myenteric plexuses revealed a slight but significant decrease in PGP 9.5 (neuronal marker) immunoreactivity (*p* < 0.01) and a parallel up-regulation of GFAP (*p* < 0.05) in DNBS rats with respect to controls (vehicle; Figure 7A,B, respectively). The increased expression of GFAP caused by DNBS was significantly reduced after *E. sativa* DSM repeated administration (Figure 7B; *p* < 0.05 with respect to the DNBS + vehicle group), though significant changes in the myenteric plexus PGP 9.5 expression were still detectable (Figure 7A), attesting that the neuronal damage is not prevented by the treatment. The above-described effects can be appreciated in the illustrative images in Figure 7C.

## 4. Discussion

The results obtained in the present work demonstrated the beneficial effects of employing *E. sativa* DSM in the treatment of colitis-associated persistent abdominal pain and intestinal damage. *E. sativa* DSM was able to acutely relieve pain in DNBS-treated animals and, by repeated treatment, to partially reduce the entity of visceral hyperalgesia persisting after colitis remission. The acute anti-hyperalgesic effect induced by the meal was mediated by the release of H_2_S and by the positive modulation of Kv7 potassium channel activity in vivo. On the other hand, the protective effect of *E. sativa* DSM on post-inflammatory pain was associated with a significant improvement in tissue recovery processes, characterized by a lower density of MCs throughout the colon and a reduced activation of the enteric glia in the myenteric plexus.

Chronic visceral pain is a disabling symptom related to IBDs and represents a therapeutic problem due to the lack of effective and safe treatments [3]. Numerous factors contribute to the maintenance of visceral hypersensitivity caused by intestinal damage, including alterations in the gut epithelial barrier, dysbiosis, immune response, and changes in neuronal signaling [35]. The multiplicity of mechanisms involved in chronic visceral pain pathogenesis makes current drugs aimed at a single target ineffective. It was thus necessary to identify a new product acting in a multi-target manner. GSLs, along with their hydrolysis products, isothiocyanates (ITCs), possess a wide range of beneficial activities, such as anti-inflammatory, probiotic, neuroprotective, and analgesic properties [7], which make them ideal candidates in the management of such a complex pathological condition. Interestingly, natural and synthetic ITCs have been reported to be highly effective in relieving either inflammatory or neuropathic pain in different preclinical models [4,6]. *Eruca sativa* DSM was conceived as a functional food able to modulate the release of GSL’s degradation products and to maximize their beneficial effects [14,36,37].

In the preclinical model of colitis induced by DNBS in rats, the acute administration of *E. sativa* DSM showed a dose-dependent pain-relieving effect. However, by pre-incubating *E. sativa* DSM suspension with Myr, which can hydrolyze the GSLs to ITCs, resulted in a lower efficacy on abdominal pain. The pain-relieving efficacy of *E. sativa* DSM might be affected either by the degradation of the contained GSLs or by the excessive number of ITCs released. This evidence is partially in contrast with our previous findings, where the same preparation was effective for diabetic neuropathic pain only after bioactivation by Myr. However, the effect of *E. sativa* DSM on visceral pain seems to be still attributable to GSLs content since it was significantly reduced by the oxidative cleavage of the GSSG disulfide moiety mediated by the chemical group ITC characteristic of GSLs derivatives [38], a reaction that prevents the release of H_2_S from ITC and the consequent anti-hyperalgesic effects, as previously demonstrated in other preclinical models of chronic pain [4,36]. The acute pain-relieving effect of *E. sativa* was also prevented by blocking Kv7 potassium channels, as observed with other natural GSLs and ITCs, and, more extensively, with all the compounds able to release H_2_S in vivo [36]. By adding Myr to *E. sativa* DSM, we are mainly delivering in vivo the ITC erucin, which has been demonstrated to act as an H_2_S donor [37] and to be responsible for the neuropathic pain relief exerted by *E. sativa* DSM in diabetic mice [4]. Pre-incubating the meal with Myr, making available in a short time a large number of ITCs, might cause a massive release of H_2_S, which can turn out to be an irritant for the gastrointestinal mucosa. Besides, ITCs might be absorbed in the first portions of the intestine and might not adequately reach their site of action in the colon. The efficacy of H_2_S donors strongly depends on their pharmacokinetic profiles—slower releasers commonly show a higher efficacy [5,39,40]. Altogether, these observations strengthen the hypothesis that H_2_S is responsible for *E. sativa’s* visceral anti-nociceptive effect.

Distrutti et al. noticed that treating rats with H_2_S dose-dependently attenuates colorectal-induced nociception by activating KATP channels [9] or by the transactivation of opioid receptors [9,10]. Similarly, the oral administration of H_2_S-releaser was found to reduce the nociceptive response of animals to colorectal stimuli [11]. H_2_S has been reported to downregulate colonic mesenteric afferent sensitivity by a nNOS-dependent mechanism [41], as well as to modulate pain signaling at the spinal dorsal horn level, ameliorating visceral pain in patients affected by IBS [39]. Nevertheless, it is important to take in account that high levels of luminal H_2_S can play a pronociceptive role in the mouse colon [42] by the activation of both Ca(v)3.2 and TRPA1 channels [40,43], confirming the advantage of employing slow H_2_S donors also in the treatment of gut pain. Among the wide range of activities played by H_2_S in different biological systems [36,44], the activation of Kv7 potassium channels was found to be the main mechanism underlying the abdominal pain relief mediated by *E. sativa* DSM, which was hampered by pretreating the animals with the Kv7 channel blocker, XE991 [4]. Interestingly, both peripheral and central Kv7 channels were found to regulate visceral sensory function in mice and humans [45,46,47]. Recently, a role for K_V_7 channel in the neuronal regulation of Cl^−^ secretion by colonic epithelium was also proposed, supporting the therapeutic potential of Kv7 channels modulators in treating pathologies associated with an hyperexcitability of enteric sensitivity terminals [48].

In this regard, it is important to make a distinction between the mechanisms responsible for the effects observed after the acute administration and those observed after the repeated administration of *E. sativa DSM*, mainly the promotion of colon healing. Indeed, behind the modulation of pain signaling, H_2_S has been reported to favor the resolution of colitis in rats [49,50], restoring intestinal microbiota biofilm and mucus production [50]. Moreover, Matsunami et al. suggested that Ca(v)3.2 -dependent stimulation of sensory neurons by H_2_S may contribute to the cytoprotection of colonic mucosa during the acute phase of TNBS-induced colitis in rats [51]. It is thus likely that H_2_S released by the GSLs contained in E. sativa DSM has a relevance in the protective effect on the gut observed after repeated treatment with the meal, though further studies are needed for elucidating this aspect. On the other hand, H_2_S-mediated effects cover a portion of the mechanisms by which GSLs and the other components of *E. sativa* DSM exert their beneficial effects on the gut. Other mechanisms may be involved in chronic pain modulation mediated by *E. sativa DSM* components [52].

GSLs have also been attributed to neuroprotective activities, since they were able to prevent the development of neuropathy induced by chemotherapy in mice [6]. Gugliandolo et al. demonstrated the anti-inflammatory and neuroprotective effects of *E. sativa,* containing GSLs and flavonoids, in an in vitro preclinical model of neuroinflammation [53]. In our case, *E. sativa* DSM did not prevent the development of visceral pain during colitis but contributed to limiting the persistence of visceral hyperalgesia after colitis resolution. This evidence highlighted the capacity of *E. sativa* DSM constituents to modify the pathophysiological processes leading to the establishment of the post-inflammatory enteric neuropathy responsible for pain consolidation. Interestingly, though *E. sativa* DSM was not able to prevent neuronal damage caused by colitis, the same treatment determined a significant attenuation of enteric glia activation within the colonic myenteric plexuses of DNBS animals. As mentioned above, enteric glial cells, which become activated during colitis in rats [34,54], are able to modulate both neuronal and immune functions and participates in visceral sensitivity regulation [18,55,56] by modulating different pathways within the gut [27,57]. *E. sativa* treatment, turning off both glia and MCs signaling, could counteract the perpetuation and the amplification of the inflammatory response, promoting tissue healing and reducing chronic pain associated with colitis, as observed in our recent study [17]. Indeed, the treatment with *E. sativa* DSM significantly reduced the number of mast cells in the colon of DNBS animals, confirming the regulation of the immune response as one of the mechanisms by which *E. sativa’s* active components promote gut healing. Notably, a dysregulation of local immune response, particularly chronic mast cell activation, is one of the mechanisms proposed to underly abdominal pain in patients with IBS [3,58,59].

Interestingly, a recent review highlighted the potential involvement of aquaporins in the GSLs and ITCs maintenance of immune homeostasis, suggesting GSLs as a novel approach to improving the symptoms in women with endometriosis [60]. Considering that many intestinal diseases are associated with changes in the aquaporin location and expression [61], the positive effect of GSLs on these channels might also contribute to the beneficial effects of *E. sativa* DSM on the gut and on gastrointestinal symptoms. Indeed, aquaporins are channels responsible for the regulation of water transport in the digestive system, which is closely related to diarrhea, constipation, and inflammatory bowel diseases [62].

Finally, it is important to mention the role played by GSLs and ITCs in the maintenance of intestinal microbial homeostasis. Recent findings attest to the beneficial effect of broths from *Lactobacillus acidophilus* fermented with *E. sativa* seed extracts on intestinal inflammation and barrier impairment caused by entero-hemorrhagic *Escherichia coli* infection [55]. GSLs and ITCs can behave akin to prebiotics by promoting lactic acid bacteria growth with a parallel increase in the levels of lactate and short-chain fatty acids [56]. Accordingly, in humans, the consumption of broccoli rich in GSLs, has been associated with both an increased abundance of *Firmicutes* and a decreased abundance of *Bacteroidetes* [58], two dominant bacterial phyla that account for 90% of the total microbial community [59]. The effect of GSLs from *E. sativa* on gut microbiota assumes a particular relevance in this work since changes in intestinal microbial composition and function have been associated with the development of several gastrointestinal diseases, such IBS and IBDs [63,64]. Noteworthy, the gut microbiota is strategically involved in the regulation of visceral sensitivity, and it has been demonstrated that microbiota-targeted interventions can counteract persistent pain resulting from colitis [16]. Although further studies are needed, the maintenance of microbial homeostasis might represent an additional mechanism underpinning the therapeutic effects of *E. sativa* DSM on the colon.

In this regard, *E. sativa* DSM might represent a therapeutic advantage both for the ease with which it can be obtained with respect to the isolated GSLs and for the synergism that might be verified among *E. sativa* components.

## 5. Conclusions

The acute pain-relieving efficacy exerted by *E. sativa* DSM on colitis-associated persistent pain, together with the protection from colon damage and pain persistence resulting from repeated administration of this preparation, supports the rational employment of *E. sativa* DSM as a nutraceutical tool for the effective treatment, both symptomatic and curative, of abdominal pain and for the restoration of gut health in patients affected by IBDs.

## Figures and Tables

**Table 1:** Chemical characterization of *E. sativa* DSM. Glucosinolates content is expressed in μmol g^−1^, total phenolic content (TPC) in mg gallic acid equivalent (GAE) g^−1^ of DSM, while total flavonoids content (TFC) in mg catechin equivalent (CE) g^−1^ of DSM. Mean values standard deviation (*n* = 3) are shown.

*E. sativa* DSM	
Glucoerucin (μmol g^−1^)	121.00 ± 2.00
Glucoraphanin (μmol g^−1^)	7.00 ± 0.30
Soluble TPC (mg GAE g^−1^)	11.00 ± 3.00
Insoluble TPC (mg GAE g^−1^)	1.33 ± 0.04
Soluble TFC (mg CE g^−1^)	4.20 ± 0.10
Insoluble TFC (mg CE g^−1^)	0.52 ± 0.08

## Data Availability

The data presented in this study are available on request from the corresponding author. The data are not publicly available due to privacy restrictions.
